# Supplementary material for: Discrimination of Methicillin-resistant Staphylococcus aureus by MALDI-TOF Mass Spectrometry with Machine Learning Techniques in Patients with Staphylococcus aureus Bacteremia
Source: Pathogens. 2022 May 16;11(5):586. doi: 10.3390/pathogens11050586 (PMC9143686; doi:10.3390/pathogens11050586)
Supplement: Supplementary file 1 [file pathogens-11-00586-s001.zip › pathogens-1661344-supplementary.pdf]

Discrimination of Methicillin-resistant *Staphylococcus aureus* by MALDI-TOF Mass Spectrometry with Machine Learning Techniques in Patients with *Staphylococcus aureus* Bacteremia

1 Genetic Algorithm

Genetic algorithm (GA) was firstly proposed by John Holland in 1975 [1]. The GA was then carried forward by the student David Goldberg of Holland. A book about GA, i.e., Genetic Algorithms in Search, Optimization, and Machine Learning, written by Goldberg is a classic book of GA, describing the basic theory of GA [2]. GA has been successfully applied in many fields, such as chemical kinetics, computer architecture, feature selection for ML, filtering and signal processing, rule set production, scheduling, learning robot behavior, image processing, molecular structure optimization, power electronics design, climatology, financial mathematics, economics, linguistic analysis, automatic design, RNA structure prediction, etc [3]. Nowadays, GA is the basic and an important algorithm in the field of evolutionary computation, a research branch of artificial intelligence.

The procedure of GA is listed as follows.

Step 0. Preliminary step: Determine the encoding method and fitness function of the chromosome. The actual encoding way and the design of the fitness function will be illustrated in Results section. In addition, it is also necessary to determine the parameter settings of GA, such as population size, the probabilities of crossover and mutation, the range of parameters to be solved, and so on.

Step 1. Generate an initial population P randomly consisting of Nchr chromosomes.

Step 2. Evaluate the quality of each chromosome through the fitness function.

Step 3. Elite policy: Pick nElt chromosomes Elt_P with the highest fitness values and store them.

Step 4. Population evolution: A new population Pnew is generated through the following genetic operations.

Step 4-1. Selection: Randomly select nC chromosomes, and generate a random number rs. If rs < p_worst (the probability of picking good or bad chromosomes), copy the individual with smallest fitness value among nC chromosomes; otherwise, copy the individual with largest fitness value among nC chromosomes.

Step 4-2. Crossover: Pick two chromosomes at random and generate a random number rc. If rs < p_crossover (probability of crossover), a two-point crossover is performed. The so-called two-point crossover is to randomly select two crossover points and swap the genes of the two chromosomes within the crossover points to produce two new chromosomes.

Step 4-3. Mutation: For each chromosome, a random number rm is randomly generated. If rm < p_mutation (probability of mutation), carry out mutation. The mutation is to randomly generate a mutation point, and regenerate the bit value for the gene at the mutation point. If the gene is encoded by binary, the 0 or 1 of the gene is reversed to 1 or 0.

Step 5. Combine the Elt_P elite chromosomes and the chromosomes obtained after mutation operation, and evaluate their fitness values.

Step 6. Select the Nchr chromosomes with the highest fitness values among the merged chromosomes [P; Elt_P] as the new population Pnew. Pick top nELt chromosomes with highest fitness values form Pnew and save them as Elt_P.

Step 7. Replace the old population P with the new population Pnew.

Step 8. If the Stop condition of the evolution has not been met (for example, a fixed number of evolutions nGen), go to Step 4 to continue the evolution; otherwise, go to Step 9.

Step 9. Choose the one with the highest fitness value from Elt_P as the best solution.

2 The design of GA for searching parameters of Support Vector Machine

The design of a chromosome and a fitness function for searching the optimal parameters of support vector machine (SVM) are introduced as follows, respectively.

(1) Chromosome design: This study uses 29 binary bits to represent a chromosome, as shown in the example in Supplementary Fig. 1. The first 12 binary bits are used to represent the parameter C. The next 12 bits are used to represent the parameter γ. The last 5 bits are used to indicate the number of features of the sample. The decoding method of parameter C and γ is to use the following formula [4]:

$$R = min_R + d\,\frac{|max_R - min_R|}{2^l - 1},$$

where R represents the actual value of the parameter; min_R and max_R indicate the minimum and maximum values of the parameter, respectively. l represents the length of the bit string, and d is the decimal value of the bit string.

(2) Fitness function definition: The fitness function *fitness* consists of two parts: weighted classification accuracy and weighted number of features as shown in below.

$$fitness = w_{acc}\, f_{acc} + w_{Nfea}\, f_{Nfea}, \text{ and } w_{acc} + w_{Nfea} = 1.$$

w_{acc} represents the weight of classification accuracy of SVM, and f_{acc} is the accuracy of 10-fold cross-validation. 10-fold cross-validation means that split the training set into 10 smaller sets. The SVM model is trained using 9 of the folds as training data, and remaining set is regarded as a testing data. Take the average of the classification accuracy of 10 testing samples as the 10-fold cross-validation performance. w_{Nfea} is the weight of the number of features. $f_{Nfea} = 1 / (N_{fea}^{1/6})$ is a

function of the number of features, where N_{fea} is the number of features in the training and testing samples.

3 Supplementary Figures and Tables

3.1 Supplementary Figures

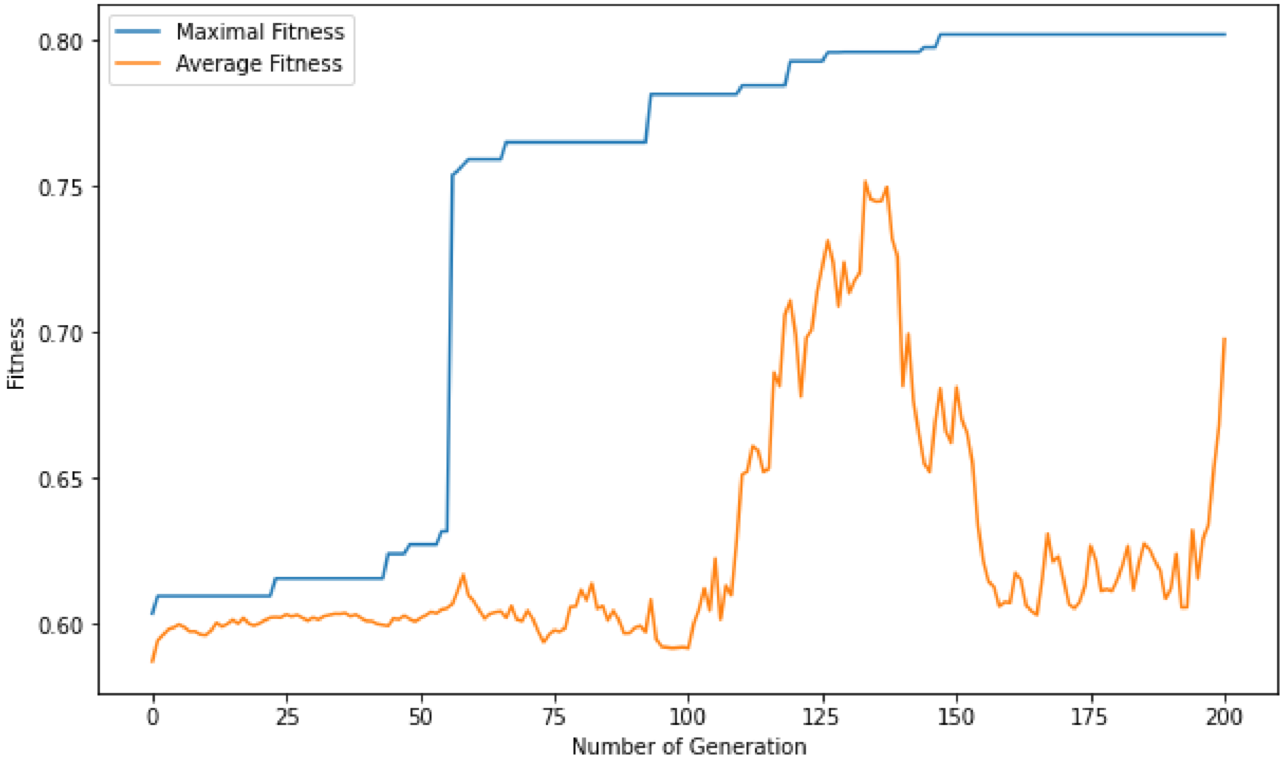

Supplementary Figure S1. The fitness values of GA during evolution for optimize SVM parameters. The maximum fitness values gradually increases, and the average fitness values oscillates randomly.

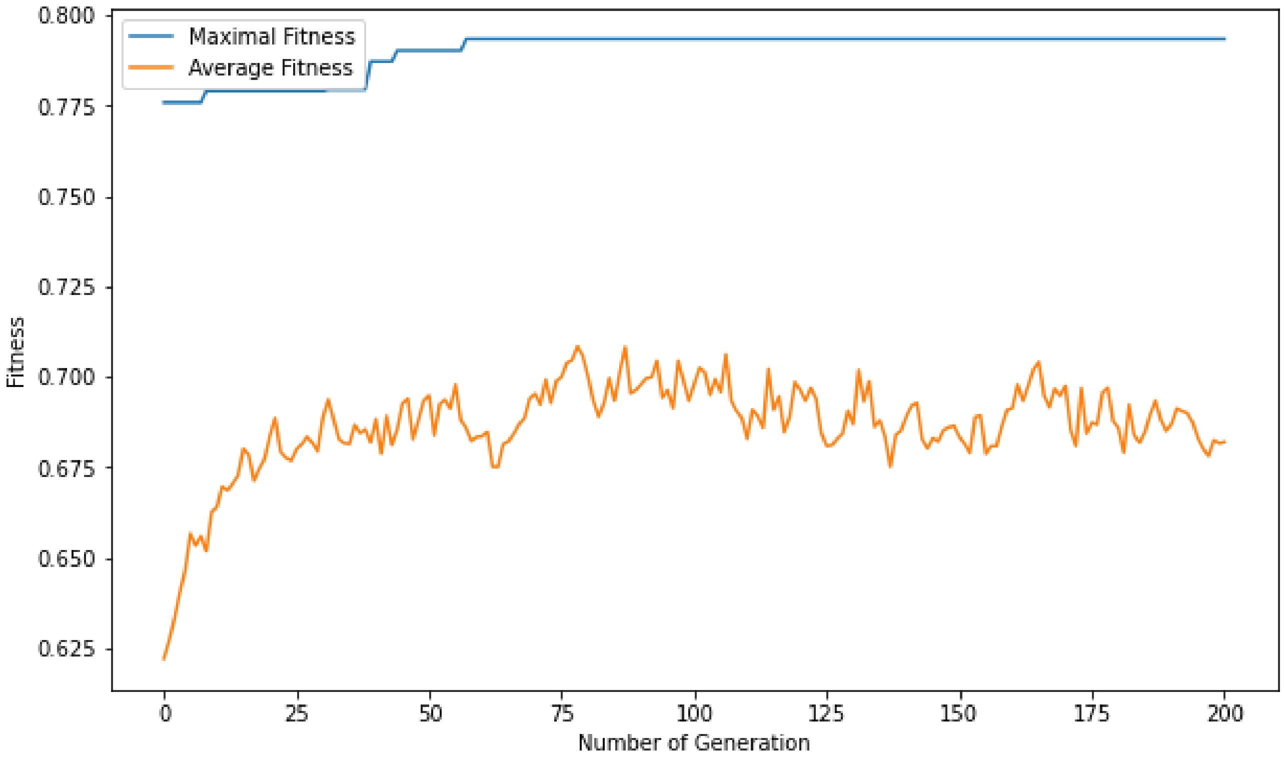

Supplementary Figure S2. The fitness values of GA during evolution for optimize DT parameters. The maximum fitness values gradually increases, and the average fitness values oscillates randomly.

3.2 Supplementary Tables

Supplementary Table S1. The classification performance of polynomial regression model in different number of features and different degrees. All important features mean all the features whose Feature Importance is greater than 0. Top 15 features mean the top 15 of all features in terms of Feature Importance. Degree *n* means that the highest power of the polynomial regression model is *n*.

Degree	Measure	Use all important features		Use top 15 features	
		Training samples	Testing samples	Training samples	Testing samples
1	Accuracy	0.8072	0.7516	0.7848	0.7638
	Sensitivity	0.7013	0.6245	0.6477	0.6242
	Specificity	0.8778	0.8399	0.8756	0.8596
2	Accuracy	1.0000	0.5559	0.8529	0.7028
	Sensitivity	1.0000	0.5289	0.7956	0.6192
	Specificity	1.0000	0.5743	0.8914	0.7588
3	Accuracy	1.0000	0.5924	0.9996	0.5147
	Sensitivity	1.0000	0.5734	0.9996	0.5120
	Specificity	1.0000	0.6061	0.9997	0.5149
4	Accuracy	1.0000	0.5801	1.0000	0.5227
	Sensitivity	1.0000	0.5492	1.0000	0.5268
	Specificity	1.0000	0.6020	1.0000	0.5207

Supplementary Table S2. The parameter settings of GA for searching optimal parameters of SVM model.

N_{chr}	N_{bit}	*p_worst*	*p_crossover*	*p_mutation*	*nElt*	*nC*
60	12	0.1	0.9	0.1	2	2
min_C	max_C	min_γ	max_γ	w_{acc}	w_{Nfea}	*nGen*
0.01	1000	0.0001	100	0.9	0.1	200

Supplementary Table S3. The parameter settings of GA for searching optimal parameters of DT model.

N_{chr}	Total bits	*p_worst*	*p_crossover*	*p_mutation*	*nElt*	*nC*	min_{MD}	max_{MD}
60	35	0.1	0.9	0.2	2	2	5	100
min_{MSS}	max_{MSS}	min_{MSL}	max_{MSL}	min_{CA}	max_{CA}	w_{acc}	w_{Nfea}	*nGen*
2	101	1	100	0	0.1	0.9	0.1	200

Supplementary Table S4. The parameter settings of GA for searching optimal parameters of RF model.

N_{chr}	Total bits	*p_worst*	*p_crossover*	*p_mutation*	*nElt*	*nC*	min_{NE}	max_{NE}	min_{MD}
30	40	0.1	0.9	0.2	2	2	50	250	5
max_{MD}	min_{MSS}	max_{MSS}	min_{MSL}	max_{MSL}	min_{CA}	max_{CA}	w_{acc}	w_{Nfea}	*nGen*
100	2	101	1	100	0	0.1	0.9	0.1	200

Supplementary Table S5. Demonstration of raw data retrieved from samples' mass spectra.

Sample	Label	peak1	peak2	peak3	peak4	peak5	peak6	peak7
S1	-1	2026.7	2041.5	2069.4	2086.0	2099.1	2167.8	2245.4
S2	-1	2037.1	2068.8	2151.7	2167.8	2244.0	2650.2	2936.4
S3	-1	2068.9	2152.0	2515.5	2651.5	2761.9	3036.6	3055.2
S4	-1	2026.4	2038.4	2069.4	2085.5	2099.3	2167.1	2185.0

Supplementary Table S6. Structured data matrix extracted with reference peaks generated by binning method.

Sample	Label	2007	2027	2038	2054	2061	2069	2085
S1	-1	0.0	2026.7	2041.5	0.0	0.0	2069.4	2086.0
S2	-1	0.0	0.0	2037.1	0.0	0.0	2068.8	0.0
S3	-1	0.0	0.0	0.0	0.0	0.0	2068.9	0.0
S4	-1	0.0	2026.4	2038.4	0.0	0.0	2069.4	2085.5

Supplementary Table S7. Data matrix presented with data values.

Sample	Label	2007	2027	2038	2054	2061	2069	2085
S1	-1	0.0	3.3	4.3	0.0	0.0	10.0	7.7
S2	-1	0.0	0.0	4.0	0.0	0.0	7.2	0.0
S3	-1	0.0	0.0	0.0	0.0	0.0	3.0	0.0
S4	-1	0.0	5.0	6.7	0.0	0.0	15.8	10.6

Supplementary Table S8. Classification performance after manually elevating feature 2410 to 2417 to the most important feature.

Based on optimal parameter settings		SVM	DT	RF	PR
Training (366 S/N samples)	Accuracy	0.8578	0.8377	0.9630	0.7779
	Sensitivity	0.8335	0.7649	0.9488	0.6279
	Specificity	0.8741	0.8865	0.9726	0.8767
Independent Testing (182 S/N samples)	Accuracy	0.8248	0.7704	0.6806	0.7449
	Sensitivity	0.7058	0.6875	0.6517	0.5965
	Specificity	0.9027	0.8246	0.6995	0.8421

References

1. Holland, J. H. (1975). Adaptation in natural and artificial systems. Ann Arbor: The University of Michigan press.
2. Goldberg, D. E. (1989). Genetic Algorithms in Search, Optimization, and Machine Learning. Reading, Massachusetts: Addison-Wesley.
3. Wikipedia contributors. (2021, September 11). List of genetic algorithm applications. In Wikipedia, The Free Encyclopedia. Retrieved 06:49, September 14, 2021, from https://en.wikipedia.org/w/index.php?title=List_of_genetic_algorithm_applications&oldid=1043678413
4. Tao, Z., Huiling, L., Wenwen, W., and Xia, Y. (2019). GA-SVM based feature selection and parameter optimization in hospitalization expense modeling. Appl. Soft Comput., 75, 323-332. doi: 10.1016/j.asoc.2018.11.001
